# Supplementary material for: Structure of LRRK1 and mechanisms of autoinhibition and activation
Source: Nat Struct Mol Biol. 2023 Oct 19;30(11):1735–45. doi: 10.1038/s41594-023-01109-1 (PMC10643122; doi:10.1038/s41594-023-01109-1)
Supplement: Supplementary file 1 — Supplementary Fig. 1. [file 41594_2023_1109_MOESM1_ESM.pdf]

nature structural & molecular biology

Article

https://doi.org/10.1038/s41594-023-01109-1

Structure of LRRK1 and mechanisms of autoinhibition and activation

In the format provided by the authors and unedited

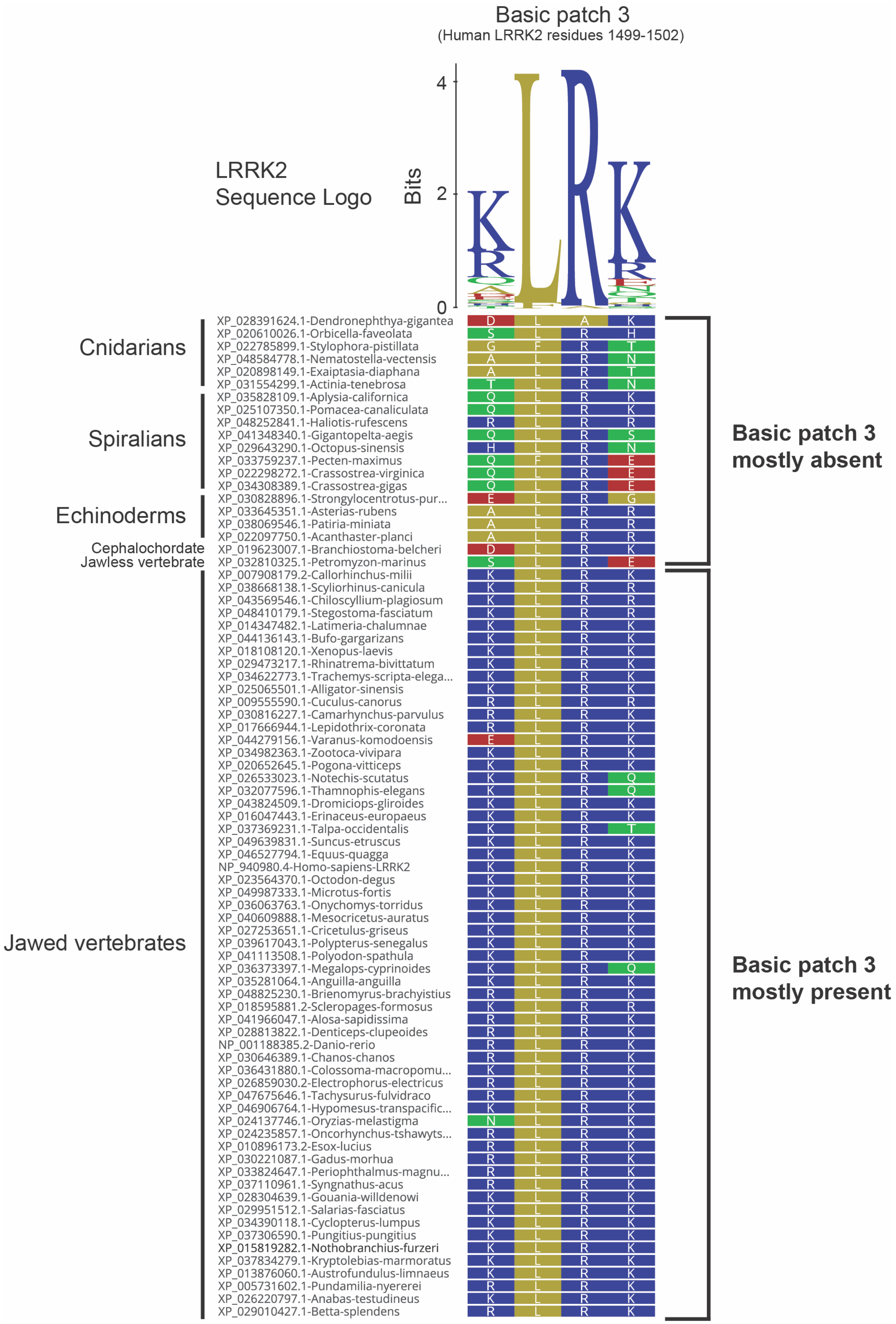

Basic patch 3
(Human LRRK2 residues 1499-1502)
LRRK2
Sequence Logo
Bits
4
2
0
Cnidarians
Spiralians
Echinoderms
Cephalochordate
Jawless vertebrate
Jawed vertebrates
XP_028391624.1-Dendronephthya-gigantea
XP_020610026.1-Orbicella-faveolata
XP_022785899.1-Stylophora-pistillata
XP_048584778.1-Nematostella-vectensis
XP_020898149.1-Exaiptasia-diaphana
XP_031554299.1-Actinia-tenebrosa
XP_035828109.1-Aplysia-californica
XP_025107350.1-Pomacea-canaliculata
XP_048252841.1-Haliotis-rufescens
XP_041348340.1-Gigantopelta-aegis
XP_029643290.1-Octopus-sinensis
XP_033759237.1-Pecten-maximus
XP_022298272.1-Crassostrea-virginica
XP_034308389.1-Crassostrea-gigas
XP_030828896.1-Strongylocentrotus-pur...
XP_033645351.1-Asterias-rubens
XP_038069546.1-Patiria-miniata
XP_022097750.1-Acanthaster-planci
XP_019623007.1-Branchiostoma-belcheri
XP_032810325.1-Petromyzon-marinus
XP_007908179.2-Callorhinchus-milii
XP_038668138.1-Scyliorhinus-canicula
XP_043569546.1-Chiloscyllium-plagiosum
XP_048410179.1-Stegostoma-fasciatum
XP_014347482.1-Latimeria-chalumnae
XP_044136143.1-Bufo-gargarizans
XP_018108120.1-Xenopus-laevis
XP_029473217.1-Rhinatrema-bivittatum
XP_034622773.1-Trachemys-scripta-elega...
XP_025065501.1-Alligator-sinensis
XP_009555590.1-Cuculus-canorus
XP_030816227.1-Camarhynchus-parvulus
XP_017666944.1-Lepidothrix-coronata
XP_044279156.1-Varanus-komodoensis
XP_034982363.1-Zootoca-vivipara
XP_020652645.1-Pogona-vitticeps
XP_026533023.1-Notechis-scutatus
XP_032077596.1-Thamnophis-elegans
XP_043824509.1-Dromiciops-gliroides
XP_016047443.1-Erinaceus-europaeus
XP_037369231.1-Talpa-occidentalis
XP_049639831.1-Suncus-etruscus
XP_046527794.1-Equus-quagga
NP_940980.4-Homo-sapiens-LRRK2
XP_023564370.1-Octodon-degus
XP_049987333.1-Microtus-fortis
XP_036063763.1-Onychomys-torridus
XP_040609888.1-Mesocricetus-auratus
XP_027253651.1-Cricetulus-griseus
XP_039617043.1-Polypterus-senegalus
XP_041113508.1-Polyodon-spathula
XP_036373397.1-Megalops-cyprinoides
XP_035281064.1-Anguilla-anguilla
XP_048825230.1-Brienomyrus-brachyistius
XP_018595881.2-Scleropages-formosus
XP_041966047.1-Alosa-sapidissima
XP_028813822.1-Denticeps-clupeoides
NP_001188385.2-Danio-rerio
XP_030646389.1-Chanos-chanos
XP_036431880.1-Colossoma-macropomu...
XP_026859030.2-Electrophorus-electricus
XP_047675646.1-Tachysurus-fulvidraco
XP_046906764.1-Hypomesus-transpacific...
XP_024137746.1-Oryzias-melastigma
XP_024235857.1-Oncorhynchus-tshawyts...
XP_010896173.2-Esox-lucius
XP_030221087.1-Gadus-morhua
XP_033824647.1-Periophthalmus-magnu...
XP_037110961.1-Syngnathus-acus
XP_028304639.1-Gouania-willdenowi
XP_029951512.1-Salarias-fasciatus
XP_034390118.1-Cyclopterus-lumpus
XP_037306590.1-Pungitius-pungitius
XP_015819282.1-Nothobranchius-furzeri
XP_037834279.1-Kryptolebias-marmoratus
XP_013876060.1-Austrofundulus-limnaeus
XP_005731602.1-Pundamilia-nyererei
XP_026220797.1-Anabas-testudineus
XP_029010427.1-Betta-splendens
Basic patch 3
mostly absent
Basic patch 3
mostly present

Supplementary Figure 1 | Presence of basic patch 3 in metazoan LRRK2s

Sequence alignment of the region of metazoan LRRK2 that corresponds to basic patch 3 (residues 1499-1502 in human LRRK2). The consensus sequence logo is shown at the top. Major species clades are shown at the left next to individual sequence accession numbers and species names. Amino acids are colored according to polarity, with basic residues shown in blue. Most jawed vertebrates have a basic-L-R-basic motif. Non-jawed vertebrate metazoans have a conserved central L-R motif, but the first and/or fourth residues tend to not be basic.
