# Supplementary material for: Structure of LRRK1 and mechanisms of autoinhibition and activation
Source: Nat Struct Mol Biol. 2023 Oct 19;30(11):1735–45. doi: 10.1038/s41594-023-01109-1 (PMC10643122; doi:10.1038/s41594-023-01109-1)
Supplement: Supplementary file 2 — Reporting Summary [file 41594_2023_1109_MOESM2_ESM.pdf]

nature portfolio

Corresponding author(s): Andres Leschziner & Samara Reck-Peterson

Last updated by author(s): 2023-2-23

Reporting Summary

Nature Portfolio wishes to improve the reproducibility of the work that we publish. This form provides structure for consistency and transparency in reporting. For further information on Nature Portfolio policies, see our Editorial Policies and the Editorial Policy Checklist.

Statistics

For all statistical analyses, confirm that the following items are present in the figure legend, table legend, main text, or Methods section.

n/a	Confirmed	
☐	☒	The exact sample size (*n*) for each experimental group/condition, given as a discrete number and unit of measurement
☐	☒	A statement on whether measurements were taken from distinct samples or whether the same sample was measured repeatedly
☐	☒	The statistical test(s) used AND whether they are one- or two-sided<br>*Only common tests should be described solely by name; describe more complex techniques in the Methods section.*
☒	☐	A description of all covariates tested
☐	☒	A description of any assumptions or corrections, such as tests of normality and adjustment for multiple comparisons
☐	☒	A full description of the statistical parameters including central tendency (e.g. means) or other basic estimates (e.g. regression coefficient) AND variation (e.g. standard deviation) or associated estimates of uncertainty (e.g. confidence intervals)
☐	☒	For null hypothesis testing, the test statistic (e.g. *F*, *t*, *r*) with confidence intervals, effect sizes, degrees of freedom and *P* value noted<br>*Give P values as exact values whenever suitable.*
☒	☐	For Bayesian analysis, information on the choice of priors and Markov chain Monte Carlo settings
☒	☐	For hierarchical and complex designs, identification of the appropriate level for tests and full reporting of outcomes
☒	☐	Estimates of effect sizes (e.g. Cohen's *d*, Pearson's *r*), indicating how they were calculated

Our web collection on statistics for biologists contains articles on many of the points above.

Software and code

Policy information about availability of computer code

Data collection	Leginon was used for all automated EM data collection. For light microscopy experiments, data was collected on commercially available Nikon Elements Software. For Western blots, data was collected using Image Studio v5.2 (Li-COR)
Data analysis	CryoSPARC Live was used to align movie frames. Particle picking was done with crYOLO. Subsequent data processing was done using Relion 3.0 and cryoSPARC.<br><br>For Western blot, data was quantified using ImageStudio v5.2. and ImageJ<br><br>Phylogenetic analysis was done using BLASTp for searching and Clustal Omega for alignment. IQ-TREE was used to generate maximum likelihood phylogenetic trees. Consensus logos and alignment visualization was done using Geneious Prime.

For manuscripts utilizing custom algorithms or software that are central to the research but not yet described in published literature, software must be made available to editors and reviewers. We strongly encourage code deposition in a community repository (e.g. GitHub). See the Nature Portfolio guidelines for submitting code & software for further information.

Data

Policy information about availability of data

All manuscripts must include a data availability statement. This statement should provide the following information, where applicable:

- Accession codes, unique identifiers, or web links for publicly available datasets
- A description of any restrictions on data availability
- For clinical datasets or third party data, please ensure that the statement adheres to our policy

All raw data that went into the biochemical and cell biological analyses were deposited in a spreadsheet with the manuscript.

Field-specific reporting

Please select the one below that is the best fit for your research. If you are not sure, read the appropriate sections before making your selection.

☒ Life sciences ☐ Behavioural & social sciences ☐ Ecological, evolutionary & environmental sciences

For a reference copy of the document with all sections, see nature.com/documents/nr-reporting-summary-flat.pdf

Life sciences study design

All studies must disclose on these points even when the disclosure is negative.

Sample size	For all experiments, we determined sample size following established conventions in the field.
Data exclusions	No data were excluded.
Replication	Rab7a phosphorylation assays by Western blot in Figures 5, 6, S3, & S4 were performed with between three and eight biological replicates. Replicate numbers for each figure are indicated in the raw data spreadsheet deposited with the manuscript. All attempts at replication were successful.
Randomization	This is not relevant. We have no data involving organisms or subjects that would require randomization.
Blinding	None

Reporting for specific materials, systems and methods

We require information from authors about some types of materials, experimental systems and methods used in many studies. Here, indicate whether each material, system or method listed is relevant to your study. If you are not sure if a list item applies to your research, read the appropriate section before selecting a response.

Materials & experimental systems

n/a	Involved in the study	
☐	☒	Antibodies
☐	☒	Eukaryotic cell lines
☒	☐	Palaeontology and archaeology
☒	☐	Animals and other organisms
☒	☐	Human research participants
☒	☐	Clinical data
☒	☐	Dual use research of concern

Methods

n/a	Involved in the study	
☒	☐	ChIP-seq
☒	☐	Flow cytometry
☒	☐	MRI-based neuroimaging

Antibodies

Antibodies used	Rabbit anti-LRRK1 antibody (ab228666), rabbit anti-GAPDH (Cell Signaling Technology, 14C10, Lot 14-2118S); mouse anti-GFP (Santa Cruz, clone: B-2, Cat: sc-9996, Lot: C1518); IRDye 800CW goat anti-rabbit (LiCOR, P/N:; 926-32211, Lot: C90229-05); IRDye 680RD goat anti-mouse (LiCOR, P/N: 926-68070, Lot: C90219-05)
Validation	All antibodies used are well-validated and highly specific commercially available antibodies. For LiCOR quantification, linear range was determined for each antibody.

Eukaryotic cell lines

Policy information about cell lines

Cell line source(s)	HEK293T used were from ATCC (CRL-3216)
Authentication	ATCC authenticated
Mycoplasma contamination	New cell lines received by our lab are tested for mycoplasma before expanding and freezing. After thawing, each cell line is tested again. Every three months, all cells growing in the lab are tested for mycoplasma as well. The cells used in our experiments were last tested on 06/23/21 and did not contain contamination.
Commonly misidentified lines (See ICLAC register)	No commonly misidentified cell lines were used.
